# The Care of Semi-Lunatics

**Published:** 1899-01-14

**Authors:** 


Vol. XXV.—No. 642.] Edited by Sir Henry Burdett, K.C.B., and Solomon C. Smith, M.D., M.R.C.P. [Jan. 14, 1899.

### The Care of Semi-Lunatics.

The recent prosecution instituted by the Commissioners in Lunacy against Mr. Reichardt, of Ewell, for the alleged offence of unlawfully receiving and detaining for payment at his residence a lunatic, Ethel Hannah Stolterfoht, and other lunatics, the said house not having been licensed for the reception of lunatics, was perhaps, in view of the suicide of the lady first mentioned, a course which could hardly have been avoided. It will be remembered that Mr. Reichardt, who is a Bachelor of Medicine of the University of London and a Licentiate of the Society of Apothecaries, and who had formerly been assistant medical officer and pathologist at Banstead Asylum, was shown to have been in the habit of receiving into his house for treatment patients who were on the border line between sanity and insanity, patients with aggravated hysteria, and others of the class which the late Dr. Marshall Hall described as "temper cases." Among these was the unfortunate lady whose death was the immediate occasion of the prosecution, Miss Stolterfoht, who was shown to have been in many respects very eccentric, and who seems at first to have been watched with considerable care. When she appeared to be better, some relaxation of this care was permitted, with the result that she escaped from the house by a window and drowned herself. An attempt had been made to certify her as a lunatic, and Drs. Savage and Goodhart had visited her for this purpose, but they both declined to give the certificate. In a legal sense at least Miss Stolterfoht was sane, and there is no law by which a medical man, or any other man, can be prevented from receiving into his house any inmate for whom such an arrangement may be made.

The state of the law in relation to real or supposed lunatics is eminently unsatisfactory, and is a mere expression of superstitious beliefs which should long since have been abandoned. It is impossible to define either sanity or insanity, and equally so, in many instances, to decide whether a particular individual should be described as sane or insane. Drs. Savage and Goodhart were unable to say that Miss Stolterfoht was insane; the sequel of her history lends great support to the belief that she might correctly have been so considered. Experience has fully proved that the large class of people whose sanity is doubtful will often recover under medical treatment, if this be conducted away from their own homes and friends, and in circumstances favourable to the enforcement of a certain amount and kind of discipline. It has fully proved that cases which might recover under such conditions will be deprived of their only chance of improvement if they are certified as lunatics and sent to an asylum. Insanity is being brought more and more every day within the domain of pathology, and is becoming more and more a subject of attention to physicians who are unconnected with asylums. Such establishments as those of Mr. Reichardt, if properly conducted, are of great advantage to the community, and their existence should by all reasonable means be promoted and encouraged. The only objection to them is the possibility, once urged by sensational writers against private asylums, that they may be abused, and that persons may be incarcerated in them whose condition does not justify such a proceeding. It would meet every difficulty if such houses were licensed, if the admission and discharge of every patient was notified to the Commissioners, and if they had power to visit whenever they were so minded. No certificate of lunacy should be required; no stigma of insanity should be thrown upon the patients who were received for treatment. The abuses which now exist are not to be found in establishments for semi-lunatics, carefully conducted by respectable and highly skilled medical practitioners, but in the instances in which insane persons are secreted in the homes of their relatives, often cruelly treated or shamefully neglected, and, at the very best, deprived of all the chances of cure which a better system might offer to them. With regard to such cases, the Commissioners might with advantage be inquisitorial, and might with advantage be severe. Their prosecution of Mr.

Reichardt could not be productive of advantage to any human being, and, in so far as it may serve to deter others from following in his footsteps, and from incurring the dangers from which he has fortunately escaped, it can scarcely fail to be seriously detrimental to the welfare of the class of persons for whose protection it was supposed to be instituted.

